# Efficacy of Omalizumab therapy in a case of severe atopic dermatitis

**DOI:** 10.1186/1710-1492-7-S2-A33

**Published:** 2011-11-14

**Authors:** Jonathan Lacombe Barrios, Louis Paradis, Anne Desroches

**Affiliations:** 1 Centre Hospitalier de l’Université de Montréal, Department of Medicine, Service of allergy and clinical immunology, Montreal, Canada; 2 Centre Hospitalier Universitaire Sainte-Justine, Departement of paediatrics, Service of allergy and clinical immunology, Montreal, Canada

## Background

Atopic dermatitis (AD) is a common skin disease of childhood which may cause debilitating symptoms and greatly impair the quality of life of the patient and his relatives [1]. Treatment of chronic AD usually focuses on topical regimen of emolients and immunosuppressants, although systemic immunosuppressive therapy is sometimes required in more severe cases. Omalizumab is a humanized monoclonal anti- IgE antibody that binds at the high-affinity receptor (FcεRI) binding site that has revealed some potential in the treatment of severe and recalcitrant AD [2].

## Case

Here, we present the case of a 11-years-old girl who has been under treatment with Omalizumab for the past five years. The patient first presented at 2 months of age with a global and severe AD involving. She was severely atopic with total IgE levels of 121,000, mild asthma, and multiple food allergies. Treatment with oral prednisone, cyclosporin, azathioprine and intravenous immunoglobulins did not improve her skin symptoms significantly. She was hospitalised multiple times for skin infections attributed to the disease and immunosuppressive medication. Treatment with Omalizumab was initiated at 6 years of age. Four months later, SCORAD index improved significantly. Since, her follow-up has been almost free of any remarkable event and treatment with Omalizumab has been well tolerated.

## Conclusion

Omalizumab should be considered as a potential treatment in cases of severe AD resistant to classical therapy.
